# Efficient Strategies to Use β-Cationic Porphyrin-Imidazolium Derivatives in the Photoinactivation of Methicillin-Resistant *Staphylococcus aureus*

**DOI:** 10.3390/ijms242115970

**Published:** 2023-11-04

**Authors:** Nuno M. M. Moura, Xavier Moreira, Eliana Sousa Da Silva, Joaquim Luís Faria, Maria G. P. M. S. Neves, Adelaide Almeida, Maria A. F. Faustino, Ana T. P. C. Gomes

**Affiliations:** 1 LAQV-REQUIMTE, Department of Chemistry, University of Aveiro, Campus Universitário de Santiago, 3810-193 Aveiro, Portugal; x.moreira@ua.pt (X.M.); gneves@ua.pt (M.G.P.M.S.N.); faustino@ua.pt (M.A.F.F.); 2 Department of Biology and CESAM, University of Aveiro, Campus Universitário de Santiago, 3810-193 Aveiro, Portugal; aalmeida@ua.pt; 3 LSRE-LCM—Laboratory of Separation and Reaction Engineering-Laboratory of Catalysis and Materials, Faculty of Engineering, University of Porto, Rua Dr. Roberto Frias s/n, 4200-465 Porto, Portugal; eliana@fe.up.pt (E.S.D.S.); jlfaria@fe.up.pt (J.L.F.); 4 ALiCE—Associate Laboratory in Chemical Engineering, Faculty of Engineering, University of Porto, Rua Dr. Roberto Frias, 4200-465 Porto, Portugal

**Keywords:** porphyrin, photosensitizer, polyvinylpyrrolidone, graphitic carbon nitride, antimicrobial photodynamic therapy, bacteria

## Abstract

Bacterial resistance to antibiotics is a critical global health issue and the development of alternatives to conventional antibiotics is of the upmost relevance. Antimicrobial photodynamic therapy (aPDT) is considered a promising and innovative approach for the photoinactivation of microorganisms, particularly in cases where traditional antibiotics may be less effective due to resistance or other limitations. In this study, two β-modified monocharged porphyrin-imidazolium derivatives were efficiently incorporated into polyvinylpyrrolidone (PVP) formulations and supported into graphitic carbon nitride materials. Both porphyrin-imidazolium derivatives displayed remarkable photostability and the ability to generate cytotoxic singlet oxygen. These properties, which have an important impact on achieving an efficient photodynamic effect, were not compromised after incorporation/immobilization. The prepared PVP-porphyrin formulations and the graphitic carbon nitride-based materials displayed excellent performance as photosensitizers to photoinactivate methicillin-resistant *Staphylococcus aureus* (MRSA) (99.9999% of bacteria) throughout the antimicrobial photodynamic therapy. In each matrix, the most rapid action against *S. aureus* was observed when using PS **2**. The **PVP-2** formulation needed 10 min of exposure to white light at 5.0 µm, while the graphitic carbon nitride hybrid **GCNM-2** required 20 min at 25.0 µm to achieve a similar level of response. These findings suggest the potential of graphitic carbon nitride-porphyrinic hybrids to be used in the environmental or clinical fields, avoiding the use of organic solvents, and might allow for their recovery after treatment, improving their applicability for bacteria photoinactivation.

## 1. Introduction

Currently, one of the most significant public health challenges is associated with antibiotic resistance, due to the inadequate prescription and overuse of antibiotics, and the consequent growing number of infections caused by multidrug-resistant (MDR) microorganisms, which is a global health threat [1]. The lack of clean water, sanitation, inadequate infection prevention and control, and the misuse and overuse of antimicrobials promotes the spread of drug-resistant pathogens in environment [2,3,4,5]. *Staphylococcus aureus* is one of the leading causes of a wide range of severe clinical infections like bacteremia and infective endocarditis, as well as osteoarticular, pleuropulmonary, and device-related infections [6,7]. Methicillin-resistant *S. aureus* (MRSA) infections represent a significant problem in world healthcare, promoting high mortality, morbidity, and financial costs [8]. Combating these threats is a public health priority that requires a collaborative global approach across sectors.

The scientific community pointed out the methodology behind antimicrobial photodynamic therapy (aPDT) as a promising and efficient alternative to antibiotics, namely, to inactivate MDR strains. This special attention was paid to the key achievements of aPDT in the environmental and clinical fields [2,9]. The aPDT approach relies on the activation of a nontoxic photosensitizer (PS) by harmless visible light in the presence of dioxygen (^3^O_2_) to produce highly cytotoxic reactive oxygen species (ROS). Singlet oxygen (^1^O_2_), the main ROS produced, can oxidize several microbial components leading to rapid microorganism inactivation [3,10,11,12]. aPDT is an attractive option compared with conventional antimicrobials, as its efficiency is independent of the microbial MDR profile, can act against several microorganisms (e.g., bacteria, viruses, parasites, and fungi) and, more notably, does not induce the development of resistance [9].

The photodynamic action strongly depends on the structure of the PS and its ability to produce ROS. Despite some classes of organic compounds that have been explored as PSs, porphyrins and related macrocycles are at the forefront of the PSs investigated for aPDT [3,13,14,15].

Porphyrins and related macrocycles display characteristics that make them unique photosensitizing agents, such as chemical versatility, photo- and storage stability, intense absorption in the visible region, efficient photoinduced reactions with dioxygen, low toxicity, and high binding affinity to different microbial components (e.g., lipids, proteins of external structures and nucleic acids) [9,16]. Another advantage is related to the multitarget nature of the photodynamic action mediated for porphyrin-based PSs, which is responsible for the irreversible inactivation of the microorganisms, reducing their possibility of acquiring resistance [17]. 

These tetrapyrrolic macrocycles, besides their biological relevance, present a unique set of properties for a wide set of applications [18], such as water/environmental remediation [19,20,21], light-harvesting devices [22,23,24,25,26], (bio)sensing [27,28,29], (photo)catalysis [30,31,32,33,34], and as PSs in photodynamic therapy (PDT) and aPDT [35,36,37,38,39,40,41,42].

Adequately functionalized porphyrins can act as broad-spectrum antimicrobial agents after being properly activated by visible light in the presence of dioxygen, targeting pathogens such as multidrug-resistant (MDR) viruses, fungi, parasites, and bacteria [43,44,45,46]. Most of these PSs can be obtained through the post-functionalization of natural porphyrin derivatives (e.g., chlorophylls and protoporphyrin IX) recurring to hard and complex synthetic routes or by the use of synthetic porphyrins like the cationic 5,10,15,20-tetrakis(1-methylpyridinium-4-yl)porphyrin (TMPyP) and analogous, limiting the synthetic approaches available to improve PS efficiency [47,48].

The possibility of extending the aPDT approach to other easily accessible synthetic porphyrinoids can be limited by their tendency to aggregate in aqueous media due to their hydrophobic features, resulting in the low production of ^1^O_2_ and consequently limiting their potential to be used as PSs. Therefore, it is important to develop adequate approaches capable of overcoming these limitations.

Considering this, we describe two efficient strategies for using non-water-soluble porphyrins as PSs in aPDT. These approaches were developed using two β-modified porphyrin-based PSs bearing imidazolium moiety and involved the incorporation of both β-modified porphyrins into polyvinylpyrrolidone (PVP) formulations and their noncovalent immobilization into graphitic carbon nitride (*g*-C_3_N_4_, GCN) materials. The photosensitizing ability of such porphyrin-imidazolium derivatives, as well as of the PVP formulations and GCN-based materials that were prepared, was assessed against MRSA strain. The results obtained disclose the potential of both PVP formulations and *g*-C_3_N_4_-based materials to act as PSs in aPDT, inducing an outstanding decrease in the viability of MRSA. 

## 2. Results

### 2.1. Photosensitizers: Preparation and Characterization

**Figure 1:**
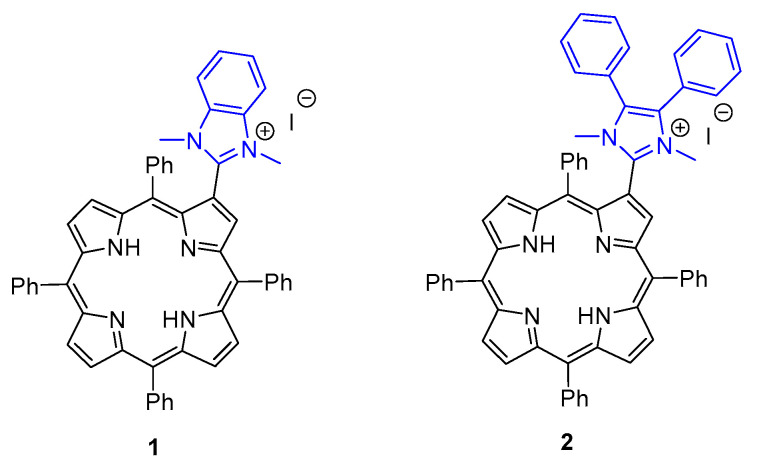
Molecular structures of the mono-charged porphyrin-imidazolium derivatives **1** and **2** (**PS 1** and **PS 2**).

The mono-charged porphyrin-imidazolium derivatives **1** and **2** (Figure 1) used as PSs were prepared by a two-step synthetic approach according to previously published procedures [49,50]. Briefly, it involved the preparation of the corresponding neutral porphyrin-imidazole derivatives throughout a Radziszewski-type reaction, followed by the alkylation/cationization of the imidazole unit to afford the desired mono-cationic PS **1** and **2**. The structures of the target mono-cationic compounds and the corresponding neutral scaffolds were confirmed by ^1^H-NMR spectroscopy and ESI(+)-MS spectrometry [49,50].

Our previous reports [49,50] revealed the potential of both β-modified porphyrins bearing imidazolium units to produce singlet oxygen and, consequently, to act as PSs to inactivate pathogenic microorganisms namely *Escherichia coli*. However, a significant drawback of these PSs is their low solubility in aqueous media, which limits their use in clinical and non-clinical applications. Aiming to surpass the solubility limitation in aqueous media, the aPDT efficacy of these β-modified porphyrins was evaluated after being incorporated into polyvinylpyrrolidone (PVP) and after being immobilized in graphitic carbon nitride supports; the results were compared with the ones without incorporation/immobilization. 

### 2.2. Incorporation into PVP Formulations

**Scheme 1:**
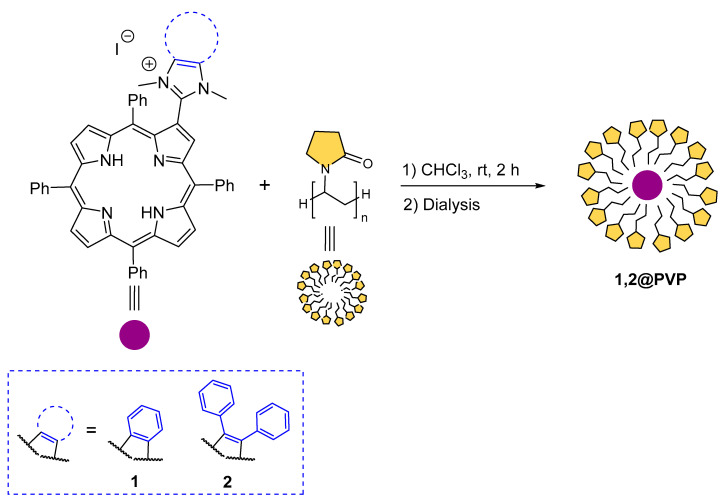
Preparation of porphyrin@PVP formulations.

PS **1** and **2** were incorporated into polyvinylpyrrolidone (PVP) formulations as outlined in Scheme 1. PVP is a nontoxic, water-soluble polymer prepared from the polymerization of the monomer *N*-vinylpyrrolidone and is widely used to improve the water solubility and pharmacokinetic and pharmacological properties of several biologically active compounds [51,52]. It is important to note that the quantities of PVP used in medical applications are not comparable to those used in food industry, and new strategies are being developed to solve its biodegradation issue [53,54,55]. Moreover, PVP formulations have been an important tool in improving the photodynamic efficiency of several photosensitizers and have been shown to be highly biocompatible and non-cytotoxic for human cell lines [56,57,58,59]. Although PVP has been used as a drug delivery system for several antibiotics and antifungal agents [60,61], to our knowledge, this strategy was not yet fully explored in regard to improving the usefulness of aPDT mediated by mono-charged porphyrins functionalized at β-positions. Most studies using PVP as a carrier system for porphyrin-based PSs are related to photodynamic processes toward cancer lines [56,62,63,64]. In fact, we also exploited this strategy efficiently to solubilize neutral β-modified porphyrin-triazole derivatives in an aqueous medium and evaluate their properties as PSs towards HT-1376 bladder cancer cells [65].

Porphyrin@PVP formulations were prepared by dissolving both PVP and the appropriate PS **1** or **2** (10% *w*/*w*) in chloroform, the mixture was stirred for 2 h at room temperature, and the solvent was removed by blowing a stream of nitrogen gas over the solution (Scheme 1). Then, the residue was taken in water and submitted to dialysis to obtain the expected formulations **PVP-1** and **PVP-2**.

**Figure 2:**
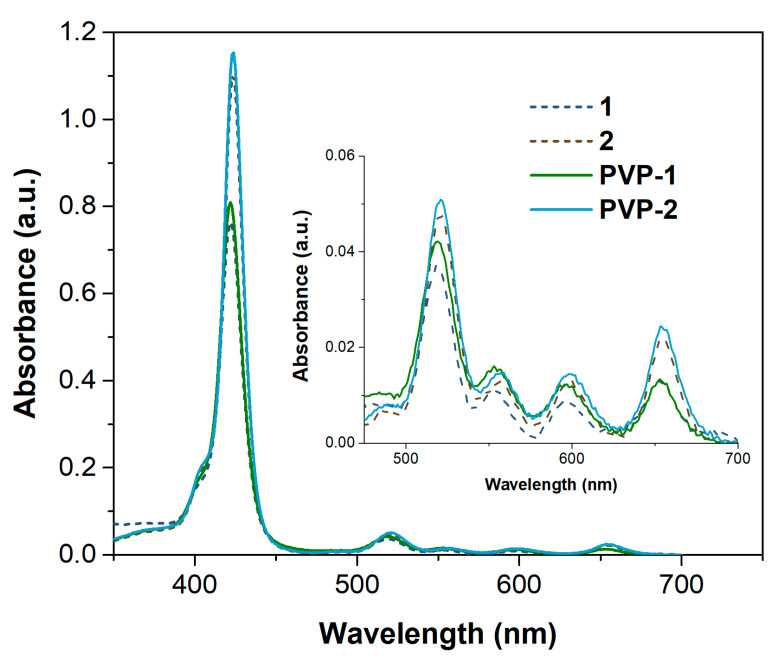
UV-Vis spectra of porphyrin-imidazolium derivatives **1** and **2** (dashed lines) and formulations **PVP-1** and **PVP-2** (solid lines) in DMF at 298 K ([**1**] = [**2**] = [**PVP-1**] = [**PVP-2**] = 5.0 × 10^−6^ M). The inset shows the absorption in the Q bands region.

Figure 2 shows the UV-Vis spectra of compounds **1** and **2** in DMF before and after their incorporation in **PVP**. As observed, the absorption spectra of compounds **1** and **2** were not affected by their incorporation in PVP formulations keeping the typical features of *meso*-tetraarylporphyrins [66,67] with a strong Soret band at 422 and 424 nm for compounds **1** and **2**, respectively, due to the π-π* transitions. Also, no noticeable changes were observed in the Q bands region ranging from 515 to 655 nm, attributed to the allowed S_0_ → S_1_ transitions. Therefore, the sharp Soret band observed for both compounds in DMF before and after their incorporation into PVP reinforces the absence of aggregates formation [67].

### 2.3. Immobilization into g-C_3_N_4_ Materials

Graphitic carbon nitride, *g*-C_3_N_4_ (GCN), is a 2D material mainly composed of N and C, based on tri-*s*-triazine and heptazine moieties [68]. In recent years, research studies using GCN-based materials have experienced a significant enhancement due to their impressive wide range of properties, such as metal-free composition, facile synthesis from low-cost precursors (urea, dicyandiamide, melamine, etc.), thermal, physical, and chemical stability, visible light absorption, and environmentally friendly nature. These features allow for applications in several fields, such as photocatalysis, environmental remediation, sensing, medicine, and optoelectronics [31,68,69,70,71,72,73,74,75]. Concerning medicinal uses, GCN-based materials were already exploited as PS agents due to their light-responsive behavior and ability to generate ROS, mainly O_2_^•−^ and HO^•^ radicals [76,77,78,79,80,81].

**Figure 3:**
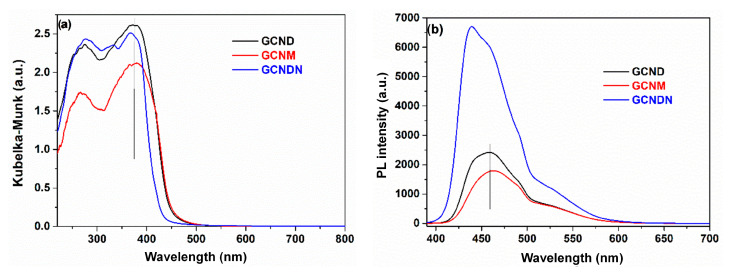
(**a**) Diffuse reflectance UV-Vis spectra and (**b**) photoluminescence spectra of **GCND**, **GCNM**, and **GCNDN** materials.

The preparation of GCN-porphyrin hybrids involved the adsorption of porphyrin-imidazolium derivatives **1** and **2** into three different GCN-based materials (two bulk and one exfoliated). All the GCN materials were prepared following well-described protocols using dicyandiamide (**D**) and melamine (**M**) as precursors to obtain the two bulk GCN (**GCND** and **GCNM**) [82]. The **GCND** material was further thermally exfoliated, giving rise to thin nanosheets (**GCNDN**) with different properties from the parent material. As illustrated in Figure 3, all the materials present absorption bands in the UV region with a maximum wavelength of around 370 nm and in the visible region up to 500 nm. However, although bulk **GCNM** has lower absorption intensity than bulk **GCND**, they present similar band edges. The opposite is observed for the exfoliated material, **GCNDN**, which shifts towards the blue. The photoluminescence (PL) spectra of the materials were also measured to obtain information regarding charge-carrier recombination. As illustrated in Figure 3b, the materials gave rise to an emission band with the maximum wavelength and intensity depending on the type of GCN precursor. Therefore, it is noticeable that **GCNM** presents a lower PL intensity, indicative of a lower recombination of electrons and holes, which is beneficial to improving photocatalytic activity. The exfoliated material, **GCNDN**, presents a higher PL intensity and a blue shift in the emission, ascribed to the confinement effect [83]. Nevertheless, the exfoliated material presented increased specific surface area (~120 m^2^ g^−1^) compared to the bulk materials (11–20 m^2^ g^−1^), which is known to be beneficial to increase the number of active sites and promote the charge carrier transfer.

**Scheme 2:**
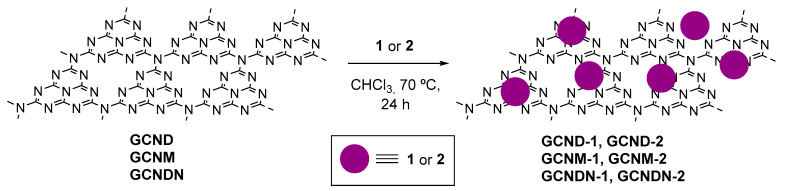
Schematic approach to prepare GCN-porphyrin hybrids.

Then, derivatives **1** and **2** were immobilized as outlined in Scheme 2 into the surface of the GCN-based materials affording the corresponding porphyrin hybrids **GCNX-1** and **GCNX-2** (where **X** stands for **D**, **M** or **DN**) containing 10% *w*/*w* of the appropriate porphyrin-imidazolium derivative (Scheme 2). It is an easy, accessible, and efficient method to prepare GCN-porphyrin hybrids through noncovalent π–π* interactions, retaining the properties of both the GCN and the porphyrin derivatives, allowing a symbiotic effect in the aPDT process.

**Figure 4:**
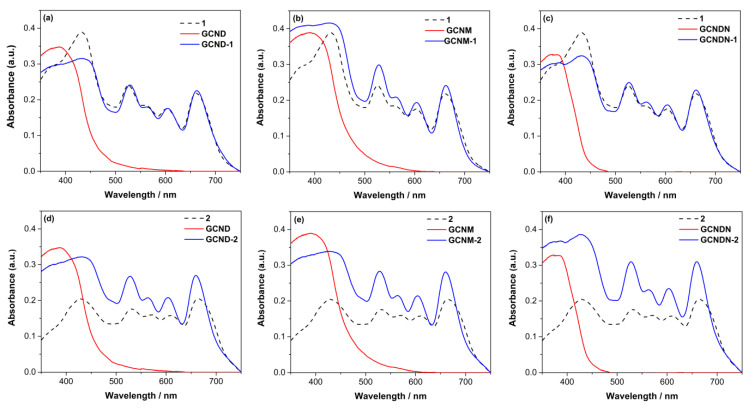
Solid-state UV-Vis spectra of GCN-porphyrin hybrids prepared by immobilization of porphyrin-imidazolium derivatives **1** (**a**–**c**) and **2** (**d**–**f**) into **GCND** (**a**,**d**), **GCNM** (**b**,**e**), and **GCNDN** (**c**,**f**), as well as derivatives **1** and **2**.

The presence of the porphyrinic derivatives at the material surfaces was confirmed by UV-Vis diffuse reflectance spectroscopy (DRS). As shown in Figure 4, GCN materials display a maximum absorption of around 370 nm, with limited absorption in visible regions above 460–500 nm. However, after the adsorption of the porphyrinic derivatives into the GCN materials, there is an enhancement in the absorption band towards ca. 475 nm, corresponding to the combination of the GCN and porphyrin Soret band absorption with a maximum of around 435 nm. The hybrids exhibit four absorption bands ranging from 528 to 663 nm due to the porphyrin Q bands ascribed to the weaker S_0_ → S_1_ transitions. The hybrids prepared with compound **1** do not show noticeable changes in both Soret and Q bands region when compared with the absorption spectrum of the mono-charge porphyrin-imidazolium **1** in solid-state (Figure 4a–c).

Nevertheless, for the hybrids prepared with derivative **2**, a 5 nm red shift for the Soret band was observed. This is accompanied by a blue shift of around 3 to 7 nm in the Q bands compared with the non-supported porphyrin-imidazolium (Figure 4a–c). These noticed changes can be attributed to the attempt of the porphyrin to amend its conformation for a more planar fashion aiming to maximize the π–π interactions with the GCN-based matrices [84].

**Figure 5:**
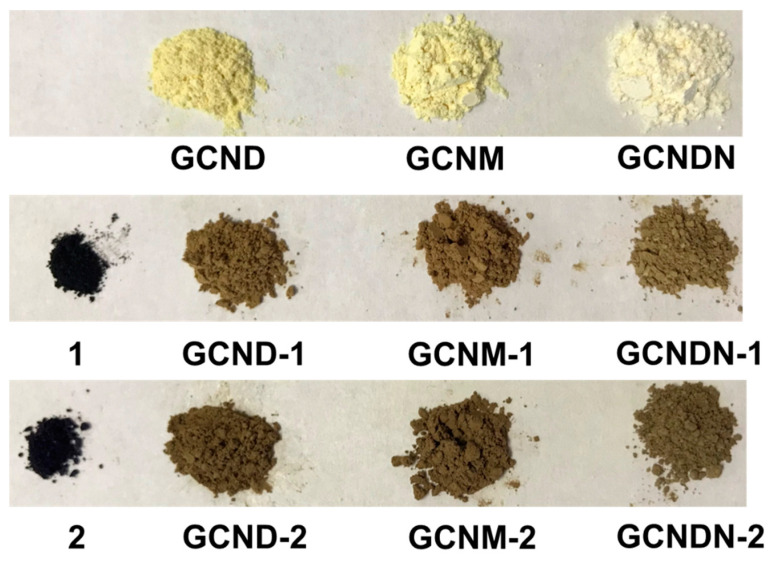
Digital photographs of the GCN-porphyrin hybrids prepared by immobilizing porphyrin-imidazolium derivatives **1** and **2**.

The changes in the solid-state UV-Vis spectra were also accompanied by a colour change at a macroscopic level of the GCN-porphyrin hybrids from light yellow to brown due to the noncovalent interactions between GCN matrices and porphyrin-imidazolium derivatives (Figure 5).

### 2.4. Photostability and Singlet Oxygen Production

The potential for compounds **1** and **2**, both PVP formulations and GCN-porphyrin hybrids, to be used as PSs in aPDT is strongly dependent on their photostability when irradiated with light, and on their ability to generate highly reactive cytotoxic species such as singlet oxygen (^1^O_2_), since the photodynamic efficiency relies on both properties [85,86].

**Figure 6:**
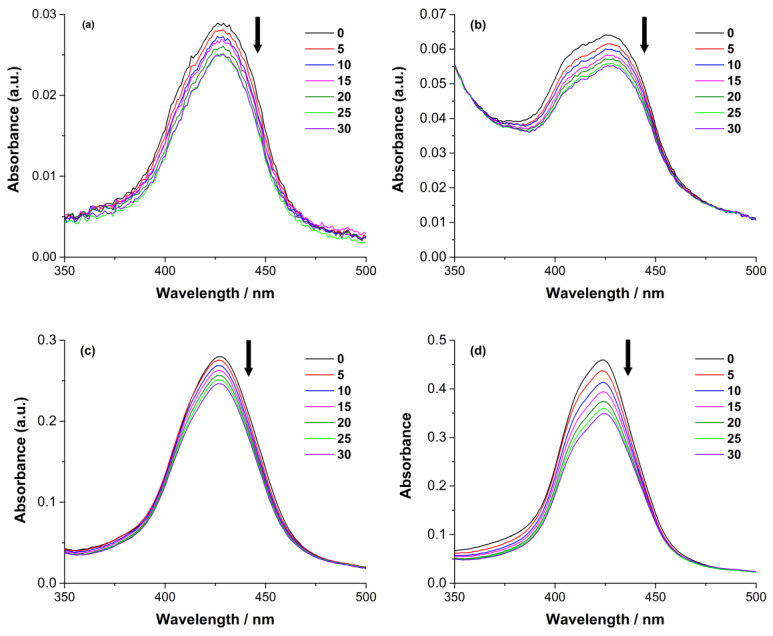
Photostability of (**a**) **1**, (**b**) **2**, (**c**) **PVP-1** and (**d**) **PVP-2** in PBS measured at the Soret band maximum after irradiation with white light at an irradiance of 40 mW·cm^−2^ for different periods of time (0–30 min) ([**1**] = [**2**] = 5.0 µM; ([**PVP-1**] = [**PVP-2**] = 5.0 µM).

**Figure 7:**
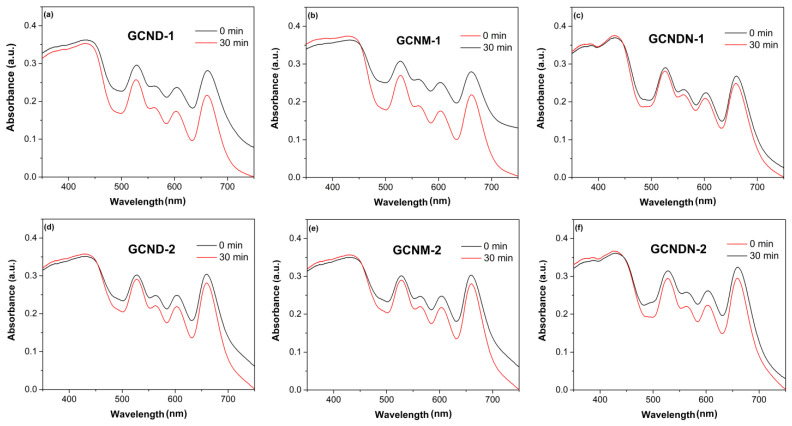
Photostability of GCN-porphyrin hybrids (**a**) **GCND-1**, (**b**) **GCNM-1**, (**c**) **GCNDN-1**, (**d**) **GCND-1**, (**e**) **GCNM-1**, (**f**) **GCNDN-1** (25 µM) after irradiation with white light at an irradiance of 40 mW·cm^−2^ for 30 min.

Photostability assays were performed by monitoring the absorption at the Soret band maximum of **1**, **2**, **PVP-1**, and **PVP-2** in PBS after irradiating with white light (370–700 nm) at an irradiance of 40 mW·cm^−2^ for 30 min (Figure 6). Porphyrin-imidazolium derivatives **1** and **2**, and the **PVP-1** formulation experienced an absorption decay at the Soret band maximum of around 15% after irradiation. In contrast, the **PVP-2** formulation displayed a decrease of ~25% at the end of the photostability assay. In all cases, the measurement of the residual absorbance at the Soret band, before and after irradiation, shows that compounds **1** and **2** and the corresponding PVP formulations are relatively photostable. To evaluate the photostability of GCN-porphyrin hybrids, an analogue approach was performed. However, since the GCN-based materials are water-insoluble, their solid-state UV-Vis spectra were recorded before and after 30 min of irradiation (Figure 7), aiming to avoid experimental errors due to the heterogeneousness of the suspension. After irradiation, a slight variation in the absorption (ca. 5%) of all the GCN-porphyrin hybrids was observed, although the smallest variation was observed with **GCNM-1**. Moreover, the lower absorption variation in the Soret band seems to indicate that **1** and **2** are more stable when immobilized in GCN materials than when incorporated in PVP formulations.

The non-significant photobleaching is a relevant parameter once it demonstrates that the PSs in solution or when incorporated in PVP formulations or supported in GCN-based materials are photostable, confirming their potential to be used in photodynamic approaches. 

The production of ^1^O_2_ was evaluated qualitatively through the first-order decay of the ^1^O_2_ quencher 1,3-diphenylisobenzofuran (DPiBF) observed at 415 nm during the irradiation of the PS in the presence of dissolved dioxygen (^3^O_2_); the DPiBF traps the resulting ^1^O_2_ in a [4 + 2] Diels–Alder like reaction affording the colorless *o*-dibenzoylbenzene [87,88,89,90]. There is consensus among the scientific community that the efficiency of PSs in generating ^1^O_2_ strongly correlates with their ability to photoinactivate microorganisms through photodynamic processes [9,91,92,93].

**Figure 8:**
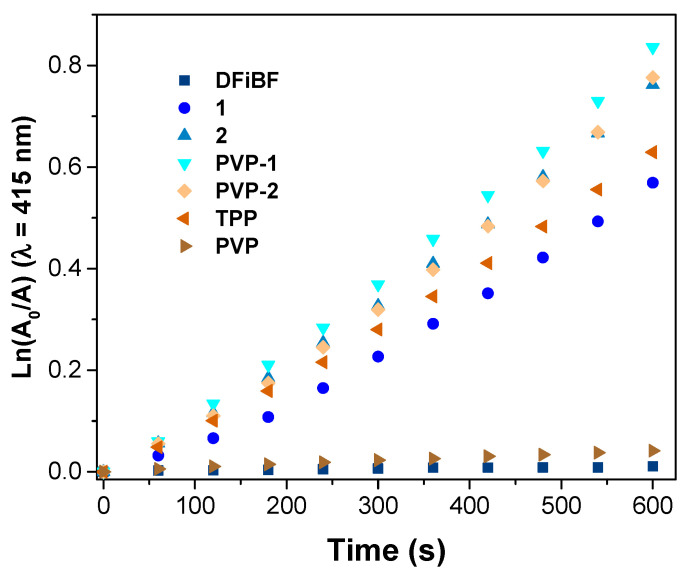
Photodecomposition of DPiBF (50 µM) photosensitized by PVP, TPP, porphyrin-imidazolium derivatives (**1** and **2**), and porphyrin-PVP-based formulations (**PVP-1** and **PVP-2**) at 0.5 µM in DMF upon irradiation with red light (630 ± 30 nm) at an irradiance of 11 mW·cm^−2^.

The assays consist of monitoring the DPiBF absorption decay at 415 nm of the irradiated DPiBF solution in DMF in the absence or in the presence of each PS before and after their incorporation in PVP (Figure 8). In general, both compounds **1** and **2** and the corresponding PVP formulations can produce ^1^O_2_, displaying high potential for use as PSs in aPDT. The **PVP-1** formulation exhibits the highest ability to generate ^1^O_2_, being around 30% higher than the reference 5,10,15,20-tetraphenylporphyrin (TPP), pointed out as a good ^1^O_2_ generator [94]. Compound **2** and the **PVP-2** formulation show a slightly lower ability to produce ^1^O_2_; even so, it is ca. 25% higher than the ability of TPP. Only compound **1** exhibited a lower production of ^1^O_2_ performance than TPP, which could be related to the higher tendency of this derivative to aggregate due to the benzoimidazolium ring at the β-pyrrolic position.

From the analysis of Figure 8, it is obvious that the absorbance of DPiBF, when irradiated in the absence of a PS as well as in the presence of just PVP, remains almost unchanged. These results reveal the relevance of the presence of the porphyrin-based PSs and prompt us to evaluate their efficiency as PSs to inactivate the Gram-positive MRSA bacterium.

It is worth mentioning that it was not possible to evaluate the ability of GCN-porphyrin hybrids to generate ^1^O_2_. Porphyrin derivatives **1** and **2** are soluble in DMF, and due to the noncovalent interaction between the porphyrinic derivatives and the GCN matrix, they will be solubilized and removed from the GCN surface. To avoid porphyrin leaching, we tried to perform the ^1^O_2_ assays in PBS solutions, but without success.

Nevertheless, since compounds **1** and **2** display outstanding performance in generating ^1^O_2_ in solution, it was envisaged that they could retain this capability when immobilized into the GCN material’s surface. Moreover, it is reported in the literature that *g*-C_3_N_4_ materials can generate ROS [77], including ^1^O_2_ [95,96,97,98]. In fact, Yang et al. [99] demonstrated that thinner GCN nanosheets produced a higher amount of ^1^O_2_. Thus, it is anticipated that GCN-porphyrin hybrids also exhibit an enhanced ability to induce microorganism death.

### 2.5. Photodynamic Inactivation of MRSA

**Figure 9:**
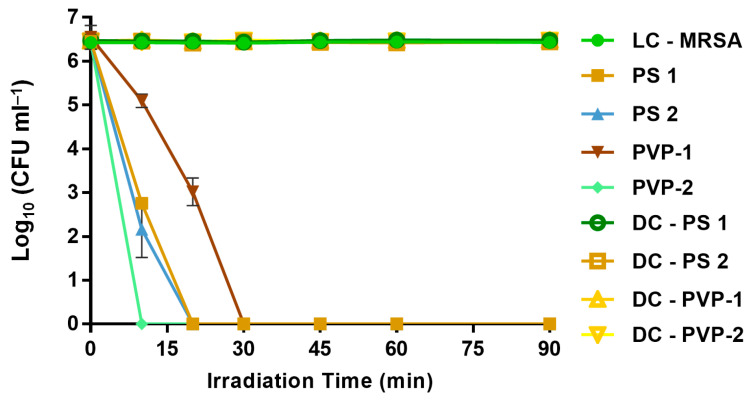
Photoinactivation profile of MRSA incubated with PS **1** and **2** and formulations **PVP-1** and **PVP-2** at 5.0 μM and irradiated with white light at an irradiance of 80 mW·cm^−2^. LC, light control; DC, dark control. Values are presented as the mean of 3 independent assays with two replicates each; the standard deviation is represented by the error bars. Lines just combine experimental points.

The aPDT efficiency of porphyrins **1** and **2** at 5.0 μM and the formulations **PVP-1** and **PVP-2** at 5.0 μM against MRSA was evaluated using white light at 80 mW·cm^−2^ for 90 min (Figure 9). The results showed the high photodynamic efficiency of **1** and **2**, displaying a similar MRSA inactivation profile, reaching the detection limit of the methodology after 20 min of irradiation (causing a decrease of 6.42 Log_10_ CFU mL^−1^, ANOVA *p* < 0.05). The formulations **PVP-1** and **PVP-2** were also efficient in the inactivation of MRSA. Formulation **PVP-2** was the most efficient formulation, promoting a decrease of 6.42 Log_10_ CFU/mL (ANOVA *p* < 0.05) in the survival of MRSA after just 10 min of irradiation. The inactivation rate profile of formulation **PVP-1** was slower, reaching the detection limit of the method after 30 min of aPDT treatment (a decrease of 6.42 Log_10_ CFU/mL, ANOVA *p* < 0.05). The lower photostability of formulation **PVP-1** could explain the difference in the photodynamic profile of the two porphyrin-PVP formulations. It is also important to note that light and dark controls showed that the MRSA cell viability was not affected by light alone (LC), neither by PSs **1**,**2** nor formulations **PVP-1**,**2** in the dark (DC), which indicates that the bacterial reduction was due to the photodynamic effect. 

**Figure 10:**
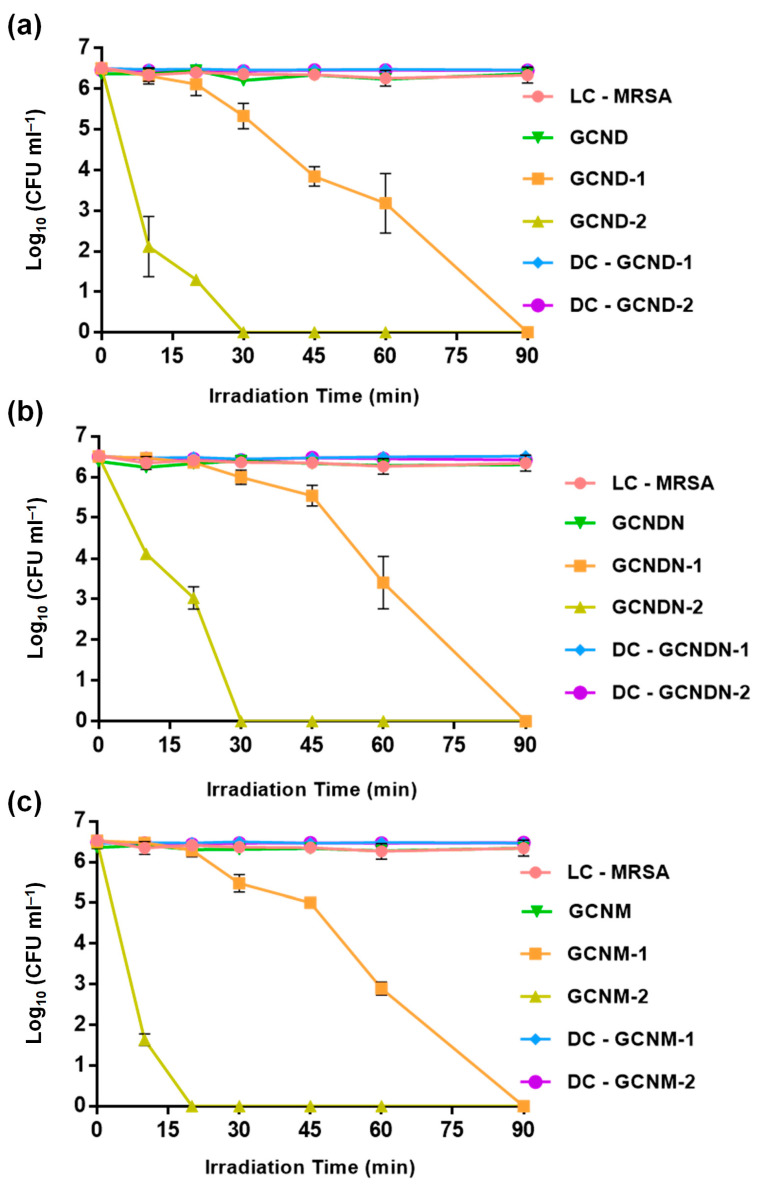
Photoinactivation profile of MRSA incubated with GCN-porphyrin hybrids (**a**) **GCND**; (**b**) **GCNDN**, and (**c**) **GCNM**) at 25 μM and irradiated with white light at an irradiance of 80 mW.cm^−2^. LC, light control; DC, dark control. Values are presented as the mean of 3 independent assays with two replicates each; the standard deviation is represented by the error bars. Lines just combine points.

The photoinactivation kinetics of MRSA promoted by GCN-porphyrinic hybrids (Figure 10a–c) show that PSs **1** and **2** can inactivate MRSA even after immobilization in these solid matrices. In general, GCN-**2** hybrids (**GCND-2**, **GCNDN-2** and **GCNM-2**) at 25 μM were the most efficient in the inactivation of MRSA, causing a decrease in the bacterium survival faster than **GCN**-**1** hybrids. For all GCN-based materials doped with PS **1**, the total inactivation of MRSA was achieved after 90 min of irradiation (i.e., a decrease of 6.40 Log_10_ CFU mL^−1^ was observed, ANOVA *p* < 0.05). However, for **GCN**-**2** hybrids, MRSA total inactivation (corresponding to a decrease of 6.40 Log_10_ CFU mL^−1^ in the MRSA survival, ANOVA *p* < 0.05) was achieved after 20 min of irradiation for **GCNM-2** (Figure 10c) and after 30 min of irradiation for **GCND-2** and **GCNDN-2** (Figure 10a and Figure 10b, respectively). These results indicate that, in general, the efficiency of the hybrids is more affected by the PS than by the origin of the GCN material. However, the response of PS **2** incorporated into the bulk GCN obtained from melamine (**GCNM**) has a faster response when compared with the ones obtained from dicyandiamide (**GCND**) (20 min versus 30 min). In all cases, LC, DC, and GCN-based materials did not promote a decrease in MRSA survival, which means that the reductions in cell viability observed in the aPDT assays of the samples treated with GCN-porphyrinic hybrids were due to the photosensitizing effect of the immobilized PS. 

These results show the enormous potential of the **PVP**-porphyrin formulations and the **GCN**-porphyrinic hybrids in the inactivation of antibiotic-resistant bacteria. There are several advantages to the incorporation/immobilization of PSs in different matrices, such as avoiding toxic organic solvents and the possibility of material reuse. Moreover, the versatility of these PSs could allow the use of this new class of PSs in the clinical area. Due to the drug delivery capability of **PVP** formulations and since these formulations avoid the aggregation of PS **1**–**2** in physiological mediums, **PVP**-**1** and **PVP-2** could be used in the disinfection of biological fluids, such as blood and plasma [100]. Moreover, **GCN**-porphyrinic hybrids, particularly the **GCNM**-based hybrid, have huge potential in treating skin infections caused by MRSA [101], where the photoactive material could be deposited on the infected skin, treated by aPDT, and then removed by a simple wash. Additionally, **GCN**-based materials already showed their high potential to be used in environmental applications, namely for hydrogen evolution and pollutant elimination, due their photocatalytic features [102,103]. The results attained in this work also open the possibility to extend the application of GCN-porphyrin hybrid materials to be used as photosensitizers for environmental purposes, namely water/wastewater disinfection.

## 3. Materials and Methods

### 3.1. General Remarks

All chemicals were utilized in their original state. Solvents underwent purification or desiccation following protocols established in the literature. For the UV-Vis absorption measurements of solutions, a Shimadzu UV-2501PC spectrophotometer (Shimadzu Corporation, Kyoto, Japan) was employed. Solid samples were subjected to UV-Vis diffuse reflectance spectroscopy (DRS) using a GBC Cintra 303 spectrophotometer (GBC Scientific Equipment Pty Ltd., Melbourne, Australia), equipped with an integrating sphere attachment. BaSO_4_ was utilized as the standard reference for reflectance. Photoluminescence (PL) spectra were recorded upon excitation at 370 nm, employing a Jasco FP-8300 spectrofluorometer (Norleq, Porto, Portugal) with a 150 W Xenon lamp as the light source.

### 3.2. Photosensitizers

Compounds **1** and **2** were synthesized using previous procedures [49,50]. UV-vis spectroscopy, ^1^H NMR, and mass spectrometry were used to confirm the structures of both porphyrin-based PS, and the spectroscopic results are in good agreement with the published results [49,50].

### 3.3. Incorporation into PVP

In a beaker, chloroform solutions of *N*-vinylpyrrolidone (PVP) and compounds **1** or **2** (10% *w*/*w*) were mixed and agitated at room temperature for 2 h to completely homogenize. The solvent was then completely evaporated using a stream of nitrogen. The resulting brownish solid was dried in an oven at 40 °C for 48 h. The resultant residues were dissolved in 1.67 mL of water and dialyzed in distilled water at pH 7 and room temperature for 24 h to provide **PVP-1** and **PVP-2** formulations.

### 3.4. Preparation of GCN and GCN-Porphyrin Hybrids

GCN bulk materials were prepared by thermal calcination as described elsewhere and were labeled as **GCND** and **GCNM** (D = dicyandiamide and M = melamine). The **GCND** material was further thermally exfoliated under air at 500 °C giving rise to a lighter and pale-yellow material labeled **GCNDN** (N = nanosheets) [82,83].

In a round-bottom flask, 100 mg of the appropriate graphitic carbon nitride derivative (**GCND**, **GCNM** or **GCNDN**) was dispersed in CHCl_3_ (5 mL) and the adequate charged porphyrin-imidazolium derivatives **1** or **2** (10% *w*/*w*) were added. The mixture was stirred under reflux for 24 h. After this period, the solvent was evaporated. The resulting powder was washed with hexane and water and dried in an oven at 40 °C for 48 h affording the desired GCN-porphyrin hybrids containing a 10% weight ratio (wt.%) of the respective porphyrin-imidazolium derivative (**1** or **2**). The presence of the porphyrin-imidazolium **1** and **2** derivatives in the GCN-based materials was confirmed by UV-Vis DRS.

### 3.5. Photostability

PBS solutions of the porphyrin-imidazolium derivatives **1** or **2** (5 µM) and PVP-based formulations **PVP-1** and **PVP-2** (5.0 µM) were prepared and stored in the dark at room temperature. The solutions were irradiated with white light (400–750 nm) for 30 min using a light-emitting diode (LED) system (ELMARK—VEGA20, 20 W, 1400 lm) at an irradiance of 40 mW·cm^−2^. Light irradiance was adjusted using a Coherent FieldMaxII top power meter combined with a Coherent PowerSens PS19Q power sensor. Absorption spectra were recorded at 0, 5, 10, 15, 20, 25, and 30 min.

For the photostability assays of GCN-porphyrin hybrids, the UV-Vis solid-state spectrum of each hybrid was recorded using diffuse reflectance. Then, each GCN-porphyrin hybrid (25 µM, the same amount used to record the solid-state UV-Vis spectra) was added to PBS and the resulting mixture was irradiated under stirring at room temperature for 30 min with the same LED system and irradiance. After this period, the solid was filtered, dried at 40 °C for 24 h, and the resulting UV-Vis diffuse reflectance spectrum was recorded.

### 3.6. Singlet Oxygen Generation Evaluation

PS (**1** and **2**) and PVP formulations (**PVP-1** and **PVP-2**) were tested for their ability to generate ^1^O_2_ by preparing stock solutions of **1** (1.17 mM), **2** (1.04 mM), **PVP-1** (3.63 mM), **PVP-2** (3.19 mM), and 1,3-diphenylisobenzofuran (DPiBF) (12.2 mM) in *N*,*N*-dimethylformamide (DMF). In a 1 × 1 cm cuvette, a 3 mL solution of PS (0.5 μM) and DPiBF (50 μM) in DMF was prepared. The solutions were irradiated for 10 min at room temperature with a red-light LED plate (630 ± 20 nm) at an intensity of 11 mW cm^−2^ and mild magnetic stirring. Control tests were also carried out, one with merely a 50 μM solution of DPiBF in DMF and the other with TPP (0.5 μM) plus DPiBF (50 μM). The absorbance decay of DPiBF at 415 nm was monitored every 1 min for 10 min.

### 3.7. PS Stock Solutions

Porphyrin-imidazolium PS **1** and **2** stock solutions in DMSO (500 μM) and **PVP-1** (3.63 mM) and **PVP-2** (3.19 mM) formulations in water were prepared and stored in the dark. Before each experiment, the solutions were sonicated at room temperature for 15 min, and depending on the concentration, a specific volume was added to the bacterial suspension.

Before each experiment, the GCN-porphyrin materials were weighed, and a specific amount of solid was added to the bacterial culture based on the desired final PS concentration.

### 3.8. Light Source

The aPDT efficiency of each PS (**1** and **2**), PVP formulations (**PVP-1** and **PVP-2**) and the GCN-porphyrin hybrids (**GCNX-1** and **GCNX-2**; where **X** stands for **D**, **M** or **DN**) was assessed by exposing the samples to white light (400–750 nm) provided by a light-emitting diode (LED) system (ELMARK—VEGA20, 20 W, 1400 lm) with an irradiance of 80 mW·cm^−2^ during 90 min. The light irradiance was adjusted using a Power Meter Coherent FieldMaxII-Top combined with a Coherent PowerSens PS19Q energy sensor.

### 3.9. Bacterial Strain and Growth Conditions

This study’s *S. aureus* strain was a methicillin-resistant strain (MRSA, DSM 25693) that produced staphylococcal enterotoxins A, C, H, G, and I [104]. MRSA was cultivated for 48 h on solid medium BD Baird-Parker Agar (BPA, Liofilchem, Roseto degli Abruzzi, Italy) at 37 °C and then stored at 4 °C. Before each aPDT experiment, one isolated colony was inoculated in 30 mL of tryptic soy broth (TSB, Merck, Rahway, NJ, USA) and cultured aerobically at 37 °C overnight for 18–24 h with 120 rpm stirring. A 300 μL aliquot of this culture was placed into a new fresh TSB liquid medium and grown under the same conditions until the stationary growth phase was reached. The aPDT assay was then performed on this culture.

### 3.10. aPDT Assays: General Procedure

The aPDT assays were carried out in accordance with Braz et al. [101]. MRSA was diluted tenfold in PBS to a final concentration of 10^7^ colony-forming units per milliliter (CFU mL^−1^) and equally distributed into the wells of a 6-well plate (6 mL per well) after reaching the stationary phase. Each PS and PVP formulation was applied to a final concentration of 5.0 M in each well. Because higher concentrations of immobilized PS are required for increased photoinactivation rates [105], GCN-porphyrinic hybrids were evaluated at 25 μM. Light and dark controls were also performed alongside with the aPDT procedure: the light controls (LC) consisted of the MRSA suspension, and the dark controls (DC) consisted of this bacterium suspension incubated with the PS or PVP formulations (both at 5.0 μM), or GCN-porphyrin hybrids (at 25 μM). To promote the engagement of each PS with the MRSA, samples and controls were incubated in the dark for 10 min with magnetic stirring before irradiation. The samples and LC were then irradiated under the specified light conditions, whereas the dark control was shielded from light throughout treatment. At 0, 10, 20, 30, 45, 60, and 90 min of light exposure, aliquots of 100 μL of samples and controls were collected and successively diluted in PBS. Dilutions were then drop-plated on Tryptic Soy Agar (TSA, Merck) using the drop plate method. The Petri plates were incubated for 18–24 h at 37 °C. The colonies were counted at the appropriate dilution, and the viable cell concentration was expressed as Log_10_ CFU mL^−1^. Three different experiments were carried out, each with two replicates. The average of the results was calculated. 

### 3.11. Statistical Analysis

GraphPad Prism 7.04 was used to access statistical analysis and normal distributions. The two-way ANOVA analysis of variance was used to determine the significance of bacterial concentration across treatments and throughout the studies. Tukey’s multiple comparison test was performed to compare the means pairwise. The significance of differences for different treatments was determined by comparing the results obtained in the samples with the corresponding control samples for the various irradiation periods. A *p* < 0.05 value was considered significant. For each assay, three separate experiments were carried out in duplicate.

## 4. Conclusions

aPDT has been demonstrating a huge potential as an alternative to antibiotics. Among the studied PSs in aPDT, porphyrins and their analogs play an important role in this process. The distinct features of porphyrinic macrocycles have encouraged several research groups to establish new synthetic routes to prepare new PSs with adequate structural, photophysical, and photochemical properties for use in aPDT. Mono-charged porphyrin-imidazolium derivatives **1** and **2** were efficiently prepared through the alkylation of the respective neutral derivatives. Incorporating these cationic porphyrin derivatives into **PVP** as well as their immobilization in graphitic carbon nitride-based materials afforded efficient PS formulations and GCN-porphyrinic hybrids for the inactivation of an antibiotic-resistant bacterium, an MRSA strain, through ^1^O_2_ generation. These findings led us to consider in the future the use of the **PVP-1,2** formulations and GCN-porphyrinic hybrids in the clinical area, namely in blood and plasma disinfection or the treatment of skin infections, and in the environmental field, in disinfection and remediation processes. 

## Data Availability

The data that support the findings of this study are available from the corresponding authors upon reasonable request.
